# Worldwide Population Genomics Reveal Long-Term Stability of the Mitochondrial Genome Architecture in a Keystone Marine Plant

**DOI:** 10.1093/gbe/evad167

**Published:** 2023-09-14

**Authors:** Marina Khachaturyan, Thorsten B H Reusch, Tal Dagan

**Affiliations:** Marine Evolutionary Ecology, GEOMAR Helmholtz Centre for Ocean Research Kiel, Kiel, Germany; Institute of General Microbiology, University of Kiel, Kiel, Germany; Marine Evolutionary Ecology, GEOMAR Helmholtz Centre for Ocean Research Kiel, Kiel, Germany; Institute of General Microbiology, University of Kiel, Kiel, Germany

**Keywords:** multichromosomal mtDNA, isoforms, chromosome stoichiometry, subgenomes, master circle, recombination, (sub)stoichiometric shifting, seagrass, eelgrass, *Zostera marina*

## Abstract

Mitochondrial genomes (mitogenomes) of flowering plants are composed of multiple chromosomes. Recombination within and between the mitochondrial chromosomes may generate diverse DNA molecules termed isoforms. The isoform copy number and composition can be dynamic within and among individual plants due to uneven replication and homologous recombination. Nonetheless, despite their functional importance, the level of mitogenome conservation within species remains understudied. Whether the ontogenetic variation translates to evolution of mitogenome composition over generations is currently unknown. Here we show that the mitogenome composition of the seagrass *Zostera marina* is conserved among worldwide populations that diverged ca. 350,000 years ago. Using long-read sequencing, we characterized the *Z. marina* mitochondrial genome and inferred the repertoire of recombination-induced configurations. To characterize the mitochondrial genome architecture worldwide and study its evolution, we examined the mitogenome in *Z. marina* meristematic region sampled in 16 populations from the Pacific and Atlantic oceans. Our results reveal a striking similarity in the isoform relative copy number, indicating a high conservation of the mitogenome composition among distantly related populations and within the plant germline, despite a notable variability during individual ontogenesis. Our study supplies a link between observations of dynamic mitogenomes at the level of plant individuals and long-term mitochondrial evolution.

**Significance**

Extensive studies on evolution of plant mitochondria in individual plants revealed great variability of the mitogenome architecture across tissues; however, data on the mitochondrion evolution at the population level are still scarce. We show that the mitochondrial genome architecture in a keystone marine plant, *Zostera marina*, remained conserved over ca. 350,000 years worldwide. We suggest that the extreme conservation of the *Z. marina* mitochondria is a manifestation of streamlined mitochondria inheritance over plant generations, for example, via a plant germline.

## Introduction

Plant mitochondrial genomes are often described as a single circular DNA molecule due to their homology to bacterial chromosomes and common convention for the description of most animal mitogenomes. However, a number of recent experiments questioned this dogma revealing a variety of multipartite mitogenomes comprising several DNA molecules (Klein et al. 1994; reviewed in Sloan 2013 and Wu et al. 2020). Mitochondrial DNA molecules can be circular, linear, or branched, where a single mitogenome can be composed of entities that vary in shape, size, and functionality (Sloan 2013; Arimura 2018; Kozik et al. 2019). Because the mitochondrial DNA is susceptible to genome rearrangements, the mitogenome assembly maps of generally large circular DNA molecules do not necessarily represent the units of inheritance or their physical state *in vivo*. Therefore, here we refer to the assembled mitogenome DNA molecules as “isoforms” as suggested in Kozik et al. 2019 (although the term “chromosome” is commonly used for any mitochondrial DNA entities; e.g., Sloan 2013; Wu et al. 2020). Most complete mitogenomes comprise up to 5 isoforms (Wu et al. 2020), yet higher numbers of up to 63 isoforms were reported in the terrestrial eudicot *Silene noctiflora* (Wu et al. 2015). Plant mitochondrial DNA molecules often contain repeated regions, which serve as homologous recombination sites. Repeats of intermediate size (e.g., 50–500 bp) have low recombinational activity, whilst large repeats are highly conducive to recombination events, hence are considered recombinationally active (Brieba 2019; Sullivan et al. 2020). Isoforms containing such recombinationally active repeats are therefore prone for recombination events that change their conformation. For example, events of reciprocal recombination via a pair of direct recombinationally active repeats in a circular DNA molecule, termed the master circle, lead to the formation of two smaller DNA molecules, termed subgenomes (first described in Palmer and Shields 1984; reviewed in Sloan 2013 and Gualberto and Newton 2017). In some plants, the multipartite mitogenome is formed solely by a single master circle and a set of corresponding subgenomes (Alverson et al. 2011; Guo et al. 2017; Dong et al. 2018). In several plant species, the mitogenome includes multiple master circles and the corresponding subgenomes (Sloan et al. 2012; Shearman et al. 2016; Shtratnikova et al. 2020). Linear mitochondrial isoforms may include telomere-like ends that are visible in the mitogenome assembly (Shtratnikova et al. 2020). At the same time, an assembled circular configuration may correspond to a combination of overlapping linear forms *in vivo*, which assist a recombination-dependent replication of the mitogenome (Gualberto and Newton 2017; Arimura 2018).

Notwithstanding the above insights, mitochondrial isoforms are still traditionally described as circular, although the predominance of circular configurations was proposed only for meristematic tissues (Arrieta-Montiel et al. 2001; Preuten et al. 2010; Woloszynska 2010; Mower, Case, et al. 2012), which are distinct from other tissues due to their contribution to the plant germline (Kwiatkowska 2008; Burian 2021). The mitochondrial main genome is thus composed of isoforms that correspond to either master circles, subgenomes, or linear configurations. In addition, occasional recombination events may lead to the formation of a dynamic variable reservoir of substoichiometric forms, which are alternative isoforms that persist in a low copy number (Small et al. 1987; Alverson et al. 2011).

Plant mitochondria constantly undergo fusion and fission that facilitate genome mixing among mitochondria within the cell (reviewed in Rose 2019, 2021). The genome intermingling may lead to individual mitochondria having an incomplete set of isoforms (Preuten et al. 2010), therefore any mitogenome should be considered at the cellular rather than the organellar level (Lonsdale et al. 1988; Rose 2021). Another notable characteristic of mitochondrial DNA is that its replication is not coordinated with cell division (termed relaxed replication; Birky 1994). One implication of relaxed replication is the possibility of an uneven replication of different mitochondrial isoforms, which may change isoform stoichiometry following cell division (Woloszynska 2010). Such changes in the relative abundance of the main genome isoforms are termed stoichiometric shifting (Gualberto and Newton 2017). In contrast, transitions between the main genome and substoichiometric forms are termed substoichiometric shifting; these occur following a dramatic increase in the relative copy number of a specific substoichiometric form, or a dramatic decrease in the relative copy number of a main genome isoform (Small et al. 1987; reviewed in Mackenzie 2007). Notably, isoform stoichiometry and its dynamics remain elusive for most thoroughly constructed multipartite mitogenomes through evolutionary time, for example, among populations of plant species.

Previous studies suggest that mitochondrial genetic variation due to (sub)stoichiometric shifting may correspond to somatic variation within individual plants (Suzuki et al. 1996; Bartoszewski et al. 2004; Sun et al. 2012; Woloszynska et al. 2012; Sloan 2013). The dynamics of recombination events and uneven replication of mitochondrial isoforms have the potential to manifest in (sub)stoichiometric shifting. To the best of our knowledge, only a single study of (sub)stoichiometric shifting at the population level was performed thus far, focusing on *S. noctiflora*; the study revealed signatures of ongoing mitogenome reduction that manifests in chromosome loss (Wu and Sloan 2019). Nonetheless, the evolution of *S. noctiflora* mitogenome may represent an extreme case of a large and highly fragmented genome; because some isoforms in *S. noctiflora* carry no genes, genome reduction due to substoichiometric shifting may be neutral. The effect of (sub)stoichiometric shifting on the evolution of plant mitogenomes thus remains understudied and requires observations of mitogenome diversity across populations. Here, we study the mitochondrial isoform stoichiometry of the marine plant *Zostera marina* (eelgrass). This species served recently as the focus of investigations on monocotyledonous adaptation to marine habitats and species radiation in the ocean (Olsen et al. 2016; Ma et al. 2021; Yu et al. 2023). *Z. marina* reproduces both sexually and via vegetative (clonal) reproduction. The latter leads to the formation of large eelgrass clones that can be several hectares in extent (Yu et al. 2020). A recent study of *Z. marina* samples collected in 16 locations worldwide revealed that all extant populations originated in the North-West Pacific and started colonizing Pacific and Atlantic oceans ca. 350 kya (Yu et al. 2023). The availability of high-resolution genomic information at the population level including a dated phylogeny enabled us to study the evolutionary dynamics of mitogenome composition of a keystone marine plant.

## Results

### *Z. marina* Mitochondrial Genome Assembly Reveals Two Major Isoforms

**Fig. 1.**
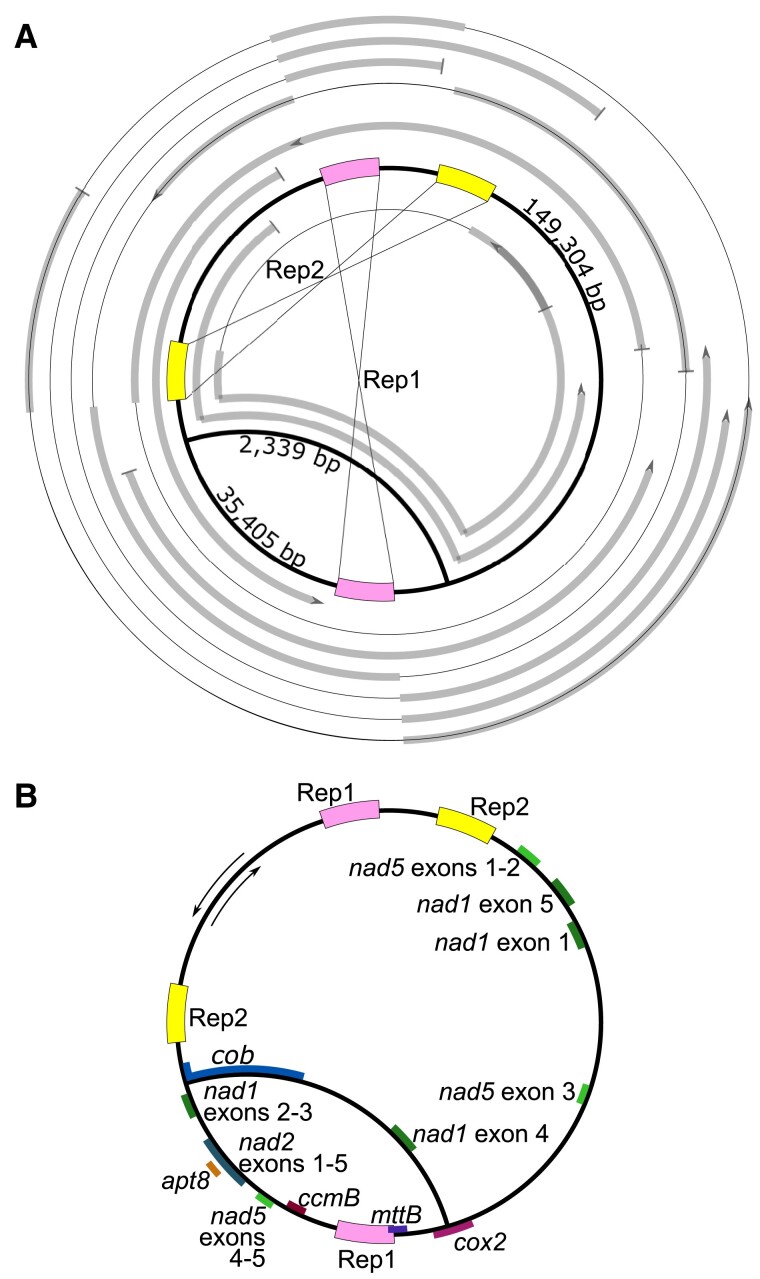
*Z. marina* mitochondrion genome assembly and annotation. (*A*) The nine mitochondrial PacBio contigs aligned to the proposed mitochondrion assembly graph. The assembly graph is depicted in thick black and the aligned contigs are depicted in thick gray. No contig parts remain unaligned. Pink and yellow boxes indicate the two largest direct repeats Rep1 and Rep2 evincing signature of recombination (thin black lines, some alignments represent an alternative flank composition of Rep1 and Rep2, which breaks the alignment into several parts). The outer contig is constructed based on a single PacBio read, hence it is unlikely to suffer from potential misassembly artifacts. (*B*) The mitochondrion assembly graph with protein-coding genes annotated at least partly on one of the two alternative edges and Rep1 and Rep2 depicted as in (*A*). Inner orientation of annotated genes reflects the positive strand, while the outer orientation represents the reverse strand genes (black arrows), and grouped exons indicate cis-splicing introns opposing to trans-splicing introns between the groups.

The *Z. marina* mitogenome sequence was assembled from nine mitochondrial PacBio contigs mapped to a three-edge assembly graph (fig. 1*A*). As the two smaller edges of 35,405 bp and 2,339 bp are alternative assembly solutions, it was impossible to reconstruct the genome as a single DNA molecule unless a duplication of 149,304 bp (the largest edge) is proposed. The presence of ambiguous edges in the assembly suggested a complex mitogenome structure with several major isoforms. As a more parsimonious solution, we therefore resolved the mitochondrial genome into two circular major isoforms iso1 and iso2 of 184,709 bp and 156,143 bp, respectively. The alternative edges are unique parts of the two isoforms and the largest edge is the common part. Because no variant sites were found in the short-read data aligned to the common part of the two isoforms, we concluded that these parts are identical. Note that the reference sample DNA was harvested from the inner layer of basal shoots in the original study (Olsen et al. 2016), therefore the presented assembly reflects the meristematic region state of the mitogenome. Our assembly of the *Z. marina* mitogenome, thus, extends the set of known multipartite plant mitogenomes that possess a unique configuration composition.

The newly assembled mitochondrion genome was annotated based on the previous annotation performed by Petersen et al. (2017). As one major difference, we found that the *cox2* open reading frame is complete rather than truncated as in the previous annotation. It is notable that the *cox2* gene is located across the transition from the common part for both major isoforms and the unique part of iso1, a feature not contained in the previous assembly (fig. 1*B*). Furthermore, our annotation includes eight additional tRNA genes. The unique part of iso2 encodes two essential genes: *cob* and *nad1-exon4*, while the unique part of iso1 encodes several seven genes: *nad1 exones2-3*, *nad2*, *apt8*, *nad5 exones4-5*, *ccmB*, *mttB*, and *cox2*. Notably, *nad1 exons2-3* and *nad1-exon4* are encoded on unique parts of the two major isoforms implying an interchromosomal trans-splicing of *nad1-intron3*, therefore both isoforms are required for Nad1 expression, such that no isoform is dominant (fig. 1*B*). The presence of all genes essential to mitochondria function, along with a set of accessory genes in the newly assembled mitogenome, serves as an internal validation for the assembly completeness.

### Recombinationally Active Repeats Manifest in Eight Isoforms

**Fig. 2.**
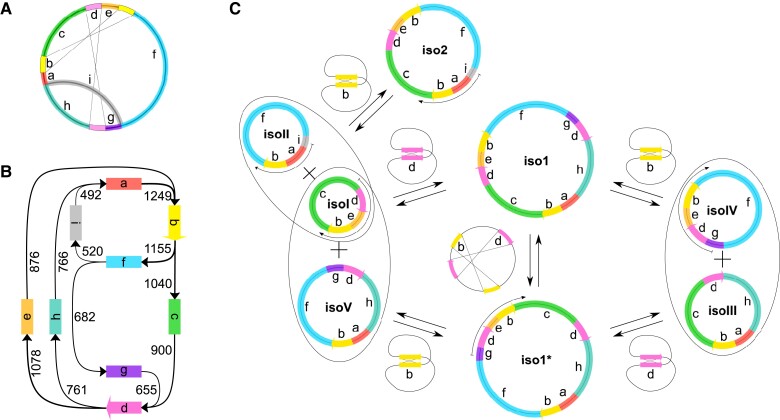
Reconstruction of mitochondrial circular main genome isoforms. (*A*) Illustration of the nine genome segments, designated by letters **a** to **i**, that remain intact during the mitogenome rearrangements via recombinationally active repeats Rep1 and Rep2. (*B*) All 12 possible genome segment connections were confirmed by PacBio reads (arrow thickness corresponds to the number of reads shown above). (*C*) Illustration of the reconstructed circular main genome isoforms. Three master circles (iso1, iso2, and iso1*) and five subgenomes (isoI-V) are depicted as circles combined by a subset of genome segments and colored correspondingly. Segment length ranges between 2,339 and 70,665 bp (see supplementary table S1, Supplementary Material online for details). Thick black arrows mark transformations resulting from reversible recombination reactions via either of the recombinationally active repeats Rep1 (pink arrow) and Rep2 (yellow arrow). Thin black arrows demonstrate examples of PacBio read alignments that confirm the corresponding configuration presence in the reference sample mitogenome. All possible configurations are composed of a subset of the five subgenomes. We note that the presence of larger circles resulting from alternative combinations of the described subgenomes cannot be ruled out.

The PacBio contig alignments to iso1 and iso2 further revealed two perfect direct repeats (Rep1—4,845 bp and Rep2—3,695 bp) and the distribution of PacBio contigs suggested that they would be recombinationally active (fig. 1*A*). A recombination event leads to an alternative sequence composition of the repeat flanking regions. The recombination activity of each repeat pair was calculated as the proportion of PacBio reads coverage of the alternative recombined composition from the total PacBio coverage of the repeat region (supplementary fig. S1, Supplementary Material online). We detected high recombinational activity of Rep1 (53.1%) and Rep2 (48.3%). Additionally, 81 repeats were characterized by a low recombination frequency (<2%) and the remaining 504 repeats demonstrated no recombinational activity. The three edges of the assembly graph in combination with the two pairs of recombinationally active repeats break the mitogenome into nine genome segments marked as **a**…**i** (fig. 2*A*). The genome segments connect in 12 possible combinations confirmed by long PacBio reads alignments (fig. 2*B*). The alternative genome segment composition indicates the existence of several different genome configurations. Based on the genome segment combinations, we reconstructed eight possible circular isoforms. The isoforms may convert into one another by homologous recombination of Rep1 and Rep2 (fig. 2*C*). Considering the possible genome transformations, isoforms iso1, iso2, and iso1* correspond to master circles, while isoI-V are regarded as subgenomes. Taken together, products of homologous recombination via Rep1 and Rep2 form a pool of eight main genome isoforms, whilst the presence of 81 repeats with low recombinational activity indicates additional substoichiometric forms.

To further validate the presence of all eight predicted isoforms, we used PacBio long-read sequencing. Each of the putative isoforms is characterized by a certain order of genome segments **a**…**i**. PacBio read alignments that cover completely the assigned combination supply a confirmation for the existence of the corresponding isoform (i.e., segment order). For example, **g-d-e-b-c** segment order appears only in iso1*, while **c-d-e-b-c** univocally defines isoI (fig. 2*C*). Using this approach, we detected at least one sustaining read for five out of eight main genome isoforms: iso1*, iso2, isoI, isoII, and isoIV (fig. 2*C*; supplementary table S2, Supplementary Material online). Notwithstanding, the expected number of covering reads depends on the segment combination total length. The remaining three configurations require considerably longer reads than those available in our data. For the same reason, iso2 and isoII were confirmed with 97 and 93 reads, respectively, whilst iso1*, isoI, and isoIV were confirmed each by 10 reads or less. The number of supporting reads points toward an equilibrium with equal amount of iso2 and isoII for the corresponding reversable reaction in the reference sample (fig. 2*C*). Our analysis, thus, confirms that the *Z. marina* mitogenome contains at least five out of eight predicted isoforms; nonetheless, their relative abundance and recombination dynamics remain elusive.

### Worldwide Population Data Reveal No Evidence for (Sub)Stoichiometric Shifting in the Eelgrass Mitogenome

**Fig. 3.**
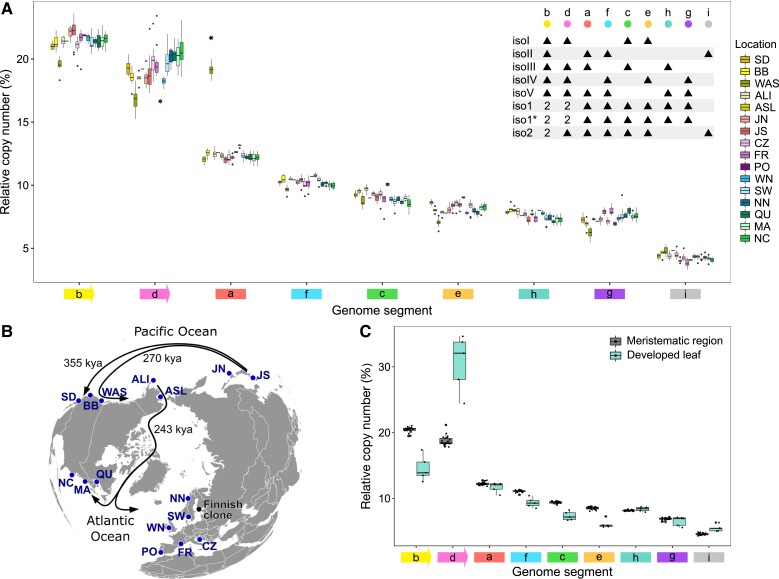
Mitochondrial isoform stoichiometry is conserved worldwide. (*A*) Relative copy number of genome segments **a**…**i** of individual samples grouped by location of sampling. Segment copy number are colored in accordance with figure 2. An increased segment copy number due to duplication in the WAS samples is highlighted by a star. Segment composition of the main genome isoforms is shown in the legend (top right) where black triangles show the presence of segments in the corresponding isoform. (*B*) Geography of all sampling locations (blue dots). Arrows indicate the three main colonization events (Yu et al. 2023). Abbreviations: San Diego, California (SD); Bodega Bay, California (BB); Washington state (WAS); Alaska-Izembek (ALI); Alaska-Safety Lagoon (ASL); Japan-North (JN); Japan-South (JS); North Carolina (NC); Massachusetts (MA); Quebec (QU); Northern Norway (NN); Sweden (SW); Wales North (WN); Portugal (PO); Mediterranean France (FR); Croatia (CZ). An additional black dot indicates the location of Finnish clone in the Archipelago Sea, southern Finland, used for meristems to leaves comparison. (*C*) Relative copy number of the genome segments **a**…**i** estimated for samples of meristematic region (*n* = 24) and developed leaves (*n* = 5) collected from the Finnish clone (Yu et al. 2020).

The occurrence of (sub)stoichiometric shifting is expected to change the relative abundance of mitochondrial isoforms over time. To investigate the evolution of isoform stoichiometry at the population level, we compared the relative mitochondrial isoform copy number among worldwide *Z. marina* populations. The analyzed data comprise genome sequences from 163 individual *Z. marina* clones sampled in 16 locations in the Pacific and Atlantic oceans (Yu et al. 2023). The sampled tissues comprised early developmental stages, that is the meristematic region and the base of first leaflets. To estimate the isoform copy number, we aligned short-read data from the populations sequencing to the reference mitogenome (as presented in fig. 2*A*) and calculated the absolute copy number of the nine genome segments (**a**…**i**). Note that the segment copy number is unaltered by the occurrence of Rep1 or Rep2 recombination (fig. 2*C*). The result revealed a similar pattern of the segment copy number in the majority of samples, regardless of the total amount of mitochondrial DNA collected, with the exception of samples having a low copy number of each segment [e.g., Alaska-Izembek (ALI) and Alaska-Safety Lagoon (ASL)] (supplementary fig. S2, Supplementary Material online). To examine the conservation of isoform stoichiometry, we compared the relative segment copy number within and among the sampled populations. Our results show that the relative copy number of the nine genome segments is homogeneous across all 16 populations (fig. 3*A*). The *Z. marina* phylogeography indicates deep divergence over 350 kya between the Californian populations and the remaining populations; another deep split between the Atlantic and Pacific populations is estimated over 240 kya (Yu et al. 2023; see fig. 3*B*). The similarity of segment copy number is thus observed across closely and distantly related populations alike. One possible trace of (sub)stoichiometric shifting may be observed in the Washington state (WAS) population. However, closer inspection of WAS sequence data (12 samples) revealed a segmental duplication in the mitogenome that altered the relative copy number of segment **a** and partially segment **h** (supplementary fig. S3, Supplementary Material online). Taken together, the similarity in segment copy number across populations suggests an unprecedent conservation of isoform stoichiometry in *Z. marina* populations worldwide.

### The Mitochondrial Isoform Stoichiometry Suggests Equal Copy Number of the Five Subgenomes

The conserved relative copy number of genome segments reported here indicates a stable stoichiometry of the *Z. marina* mitochondrial isoforms. Note that inferences on isoform stoichiometry via short-read mapping and without additional long-read data require that reversible recombination reactions are considered “fixed” (i.e., implying a certain proportion of the master circle and subgenome products). For that purpose, we first fixed the recombination reactions such that the master circles iso1 and iso2 are the only remaining isoforms. Using that assumption, the copy number of segment ***a*** and segment ***e*** is equal, because both segments are present once in each master circle. However, our data clearly suggest that segment ***a*** has a higher abundance comparing to segment ***e***. Moreover, the relative copy number of segments ***a*** and ***e*** seems to match well their presence in the subgenomes. Segment ***a*** is present in three of the subgenomes and segment ***e*** in only two subgenomes (figs. 2*C* and 3*A*; supplementary fig. S2, Supplementary Material online). Notably, the relative copy number of the remaining segments precisely reflects their expected quantity according to the subgenomes segment composition (fig. 3*A*). Consequently, we suggest to consider the five subgenomes isoI-V as primary isoforms in the eelgrass mitogenome. Conversely, the master circles iso1, iso2, and iso1* should be considered as occasional combination of certain subgenomes. Therefore, our assessment of the isoform stoichiometry focuses only on the five subgenomes isoI-V (see fig. 2*C*).

The relative copy number of the five subgenomes can be estimated from the copy number of genome segments **a**…**i**. However, a direct assessment of the relative abundance of all five isoforms is problematic due to the possible transformation of isoIII and isoIV to isoI and isoV (fig. 2*C*). For the purpose of our calculation here, we therefore set isoIV’ copy number to zero. The apostrophe symbol in the isoform names is used here when considering the isoform abundance under the assumption of recombination reaction fixations. Note that the copy numbers of segments **e**, **i**, and **g** correspond to the copy number of isoI’, isoII’, and isoV’, respectively (supplementary fig. S4*A*, Supplementary Material online). Hence, the copy number of isoIII’ can be measured as the difference in copy number of the three segment pair combinations: **h** and **g**, **c** and **e**, or **a** and **f** (see fig. 3*A* legend; supplementary fig. S4*A*, Supplementary Material online). Estimating isoIII’ copy number using the first combination, **h** and **g**, revealed that the proportion of the five subgenomes isoI’:isoII’:isoIII’:isoIV’:isoV’ slightly deviates from a ratio 2:1:0:0:2 (supplementary fig. S4*B*, Supplementary Material online). Note that under the above assumption, the fraction of isoIV’ is zero, because the copy number of the subgenome isoIV had to be set to zero for all samples. The narrow distributions of the subgenome relative abundance among the samples supply a confirmation for the conservation of isoform stoichiometry across all populations. Furthermore, if we suppose equal number of pairs isoI-isoV and isoIII-isoIV, we obtain a stoichiometry with a ratio of 1:1:1:1:1 (supplementary fig. S4*C*, Supplementary Material online). Similar observations of roughly even ratios of the mitochondrial isoforms have been made for *S. noctiflora*, *Silene conica*, *Psilotum nudum*, and *Lophophytum mirabile* (Sloan et al. 2012; Guo et al. 2017: 20; Sanchez-Puerta et al. 2017). The equality of copy number among the five subgenome in *Z. marina* would correspond to a naturally stable equilibrium.

Repeating the estimation of isoIII’ copy number using the two other segment combinations **c** and **e**, and **a** and **f**, results in a significantly higher relative copy number (supplementary fig. S4*D*, Supplementary Material online), contrary to expectations based on the segment composition of the main genome isoforms (fig. 3*A*). Even if we have to suggest an unknown component of the isoform composition, the narrow distribution of the estimated isoIII’ relative copy number using the above two combinations suggests that the source of deviation is common to all sampled populations. We conclude that the *Z. marina* mitogenome likely contains additional isoforms that are not composed of the five subgenomes isoI-V; the existence of such alternative isoforms would alter the relative abundance of certain genome segments.

### Mitochondrial Isoform Composition in Developed Leaves Differs From the Meristematic Region

The striking conservation of mitogenome isoforms may point toward either a specific property of *Z. marina* due to its compact genome or a specific property of meristematic region due to its contribution to plant germline. In order to resolve the ambiguity, we analyzed additional samples collected from a single *Z. marina* clone from the Archipelago Sea, southern Finland (Yu et al. 2020). The relative copy number of the nine genome segments was calculated for 24 samples of the meristematic region and 5 samples of developed leaf tissue (fig. 3*C*). The estimated abundance of all genome segments in the meristematic samples was within the range of the values calculated from the worldwide population dataset (fig. 3*A* and *C*). Hence, the mitogenome composition observed worldwide is conserved in the Finnish clone as well. Note that the 24 analyzed samples were collected from the same clone and thereby correspond to a single datapoint in the worldwide population dataset. In contrast, the estimated relative copy numbers in developed leaves were markedly different from those observed in the meristematic region, especially for segments **b**, **d**, **f**, **c**, **e**, and **i** with nonoverlapping distributions (fig. 3*C*). Furthermore, the leaf samples show a wider distribution of the estimated genome segment copy number, even within a single clone, indicating the occurrence of independent (sub)stoichiometric shifting events during ontogenesis. Thus, the mitogenome composition is more diverse between different plant tissues of the same clone than between meristems of populations diverged ca. 350,000 years ago.

## Discussion

Variation in isoform stoichiometry is a key characteristic of plant mitochondrial genomes where isoform copy number in mitochondria may alternate by (sub)stoichiometric shifting. A long-standing question is therefore whether and how (sub)stoichiometric shifting translates to the next generation thus contributing to the evolution of mitochondrial genetic diversity over time. Here we demonstrate that the mitochondrial isoform copy number in *Z. marina* meristematic region remains homogeneous among widely divergent populations, implying a conservation of isoform stoichiometry in the plant germline over 350,000 years since the last common ancestor of extant populations (Yu et al. 2023). Our results thus reveal a novel perspective on plant population genetics.

Quantifying the stoichiometry of products from isoform recombination reaction—that is, master circles and their subgenomes—at equilibrium remains challenging. Long-read data enabled us to characterize the pool of mitochondrial isoforms and further reveal evidence for equal copy number of the master circle iso2 and its subgenome isoII. The measured recombinational activity of Rep1 and Rep2 is close to 0.5 (supplementary fig. S1, Supplementary Material online), which furthermore points toward equal amounts of the products in all reversible recombination reactions. Our results thus differ from other studies that reported significant superiority in copy number of subgenomic molecules over the master circles (Alverson et al. 2011; Sloan et al. 2012). The relative copy number of master circles to subgenomes may vary among plant tissues and reaches the prevalence of master circles in the plant meristem, which may be essential for the mitogenome heritability (Arrieta-Montiel et al. 2001; Preuten et al. 2010; Woloszynska 2010; Mower, Sloan, et al. 2012). Our observation raises the question whether the stoichiometry of recombination reaction equilibrium shifts rapidly or rather remains stable due to certain regulatory mechanisms, for example nuclear control of homologous recombination (Albert et al. 2003; Arrieta-Montiel et al. 2009).

The conventional way for describing a mitochondrial genome is to present master circles as primary isoforms and subgenomes as occasional products of homologous recombination, even if the subgenomes eventually dominate in quantity. This approach incorporates an assumption that subgenomes can be grouped into pairs to form the master circles. However, our data support the opposite perspective: subgenomes, rather than master circles, should be considered as the relevant genetic entities, similar to observations made for the monkeyflower mitogenome (Mower, Case, et al. 2012). Here we referred to alternative DNA configurations as isoforms, because the mitogenome physical representation and its units of inheritance, namely chromosomes, remain elusive. However, the identification of relevant genetic entities might supply a hint for the possible mitochondrial chromosome composition. Our analysis of segment copy number suggests that focusing on subgenomes may still supply an incomplete mitogenome representation. The transformation of genome segment copy number into the isoform stoichiometry revealed an unexpected significant difference using different formulations (supplementary fig. S4*C*, Supplementary Material online), all based on possible rearrangements of the main genome isoforms (fig. 2*C*). This result suggests the presence of additional configurations that affect the genome segment abundance but are not represented in the main genome (i.e., these are being generally underestimated). The conventional depiction of plant mitogenomes solely as master circles and subgenomes, thus, supplies a partial depiction of the isoform composition diversity.

To achieve a complete mitogenome representation, we suggest two alternative hypotheses for the source of deviation in segment copy number from the expectation according to the repertoire of main genome isoforms. The first possible explanation to our conundrum is an effect of substoichiometric forms. Although each substoichiometric molecule persists in a much lower proportion than the main genome isoforms, the substoichiometric reservoir altogether could be responsible for the shifts in genome segment relative copy number. For example, an underrepresentation of segment **f** in substoichiometric forms would lead to an overestimation of isoIII’ copy number when using the **a–f** formulation (supplementary fig. S4*C*, Supplementary Material online). We note, however, that because the variation of **a–f** among populations is rather small, this explanation implies that the composition of the substoichiometric forms remains stable across all populations, in agreement with previous suggestions in the literature (Laser et al. 1997; Arrieta-Montiel et al. 2001; Woloszynska and Trojanowski 2009; reviewed in Woloszynska 2010). An alternative possibility is the presence of partially overlapping subgenomic linear molecules instead of, or in addition to, complete circular molecules (reviewed in Gualberto and Newton 2017; Arimura 2018). Overrepresented genome segments may thus correspond to overlapping regions of subgenomic linear molecules. For example, if such overlap of linear forms is found within segment **a**, then this will lead to an overestimation of segment **a** copy number and therefore a deviation of **a–f** from our expectation. Considering that linear molecule ends are proposed to be recombination-dependent replication start points, the small variation in **a–f** under this scenario suggests that the origins of replication are conserved throughout all populations.

The mechanisms controlling mitochondrion isoform stoichiometry remain unclear and are most likely a combination of several factors. Two mechanisms have been discussed in the literature: (i) the effect of nuclear control of recombination reaction dynamics (Albert et al. 2003; Arrieta-Montiel et al. 2009) and (ii) having isoform conformations that are specific to meristematic tissues, which may reduce the possibility of (sub)stoichiometric shifting (Arrieta-Montiel et al. 2001; Woloszynska 2010; Liberatore et al. 2016). Additional possibility is selection pressure related to change of gene dosage. Relative expression of genes located on isoforms having different abundance might have a gene dosage effect, which could be subject to stabilizing selection, similarly to observations made for prokaryotic plasmids (Nicoloff et al. 2019).

Our study reveals a striking conservation of the mitochondrial isoform composition and stoichiometry in meristematic regions across worldwide populations. Here we assumed that mitochondria are uniparentally inherited together with the chloroplasts and thereby featuring a similar phylogeny with the deepest divergence event to have occurred between the Californian and main Pacific populations over 350 kya. However, even if the mitochondrial genome is admixed along the Californian coast, the divergence between Pacific and Atlantic over 240 kya provides a solid estimate (Yu et al. 2023). This observation is all the more surprising given the complex mitogenome architecture revealed here. At the same time, developed leaf samples exhibit evidence for (sub)stoichiometric shifting in comparison to the meristematic regions of the same clone, in agreement with previous observations in *Phaseolus vulgaris* (Woloszynska et al. 2012). Given the above, we conclude that *Z. marina* germline is preserved from (sub)stoichiometric changes and therefore they are not transmitted to next generations neither vegetatively nor by sexual reproduction. Notably, our observations indirectly support the hypothesis that differentiated somatic cells rarely contribute to the plant germline by dedifferentiation. Otherwise, the shifts in mitogenome compositions that appear in somatic cells would manifest in the germline and cause significant differences in distantly related populations. Alternatively, cell dedifferentiation in plants should involve reversive changes in the mitogenome composition.

Mitogenome architecture and gene order are highly variable among plant species, even those that are closely related (Palmer and Herbon 1988; Mower, Sloan, et al. 2012). Consequently, we suggest that changes in isoform stoichiometry are not a major driver in the evolution of mitogenome architecture in eelgrass. Instead, we predict intrachromosomal rearrangements, for example, duplication, translocation, and inversions, as main events for mitogenome evolution during Zosteraceae species divergence, as observed in the WAS samples. Notably, rearrangements of the mitochondrial isoforms in *Brassica napus* lines may lead to a cytoplasmic male sterility (Chen et al. 2011), thus supporting a role of such rearrangements in speciation events. Substoichiometric shifting, in contrast, may be a plastic property of mitochondrial genomes in somatic tissues that is inhibited in the germline by specific buffering mechanisms. Our findings open up avenues for further research on the evolution of genome architecture and stoichiometry in plant mitochondria, its maintenance, as well as possible implications for plant diversification.

## Materials and Methods

### Data Source

The chromosome-level nuclear and chloroplast *Z. marina* genome assembly was downloaded through the online resource for community annotation of eukaryotes (ORCAE) platform (https://bioinformatics.psb.ugent.be/gdb/zostera/). The long-read PacBio sequencing data were downloaded from National Center for Biotechnology Information (NCBI), accession number: PRJNA701932 (Ma et al. 2021). The Illumina whole-genome sequencing of *Z. marina* meristematic region of the inner leaf base was obtained from the NCBI database, BioProjects: PRJNA41721, PRJNA462974, and PRJNA557092 for the reference sample sequencing, the worldwide population dataset, and the Finnish clone dataset correspondingly (Olsen et al. 2016; Yu et al. 2020, 2023). Worldwide population dataset samples were preselected according to Yu et al. (2023) to avoid representatives of the same clone and selfing. In addition to the published data sets of the global population-level analysis, and the 24 clone mates at Ängsö, additional plants of the Finnish clone were collected in 40 × 40 cm areas in summer 2021 by scuba diving along a horizontal transect line in 2 metre water depth at locations ca. 20 metres distant from one another. Approximately 80 mg of dried leaf tissue (deliberately excluding the meristematic region) from a total of 6–8 leaf shoots at each spot were extracted as in Yu et al. (2023) and subjected to full genome resequencing using a paired end PE150 library preparation and an Illumina HiSeq X Ten sequencing platform to a coverage of approximately 50 × at BGI Hongkong. Sequences have been submitted to NCBI Short-Read Archive (SRA) (BioProject: PRJNA972892; SRA accessions: SRR24578335–SRR24578339).

### Mitochondrial Genome Assembly

The mitogenome sequence of *Z. marina* was previously reported to include a single chromosome (Petersen et al. 2017; RefSeq: NC_035345.1). The availability of PacBio long-read sequence data that was recently used to assemble the *Z. marina* nuclear genomes to chromosomal resolution (Ma et al. 2021) enabled us to also assemble the mitochondrial genome with high accuracy. PacBio long reads were assembled into contigs by Canu (version 1.9) with default parameters and expected genome size of 230 Mbp (Koren et al. 2017). The result contigs were aligned to the mitogenome assembly from Petersen et al. (2017) by nucleotide basic local alignment search tool (BLAST) (version 2.2.28) with default parameters (Altschul et al. 1990). Contigs with over 10 kbp long alignment to the subject mitogenome, including nine contigs with the length range of 49,378–115,620 bp, were considered mitochondria-related and used for further assembly. The remaining contigs mapped to the reference mitogenome in less than 30% of their lengths suggesting that they belonged to chimeric assemblies or nuclear DNA. The assembly process was based on manual examination of the overlap between the nine contigs and resulted in the three-edge assembly graph (fig. 1). Self-duplications and breaks in several alignments are found next to the Rep1 and Rep2 repeats; self-duplications are likely to be the result of a contig misassembly, whilst the breaks may supply evidence for alternative configurations. All nine contigs could be aligned to the proposed mitochondrial genome assembly graph entirely, thereby excluding any additional ambiguities (fig. 1). The mitogenome circularity was confirmed by discontinuous coverage by PacBio contigs (fig. 1) and Illumina short reads (as suggested in Petersen et al. 2017). Moreover, the mitochondrial genome proposed in Petersen et al. (2017) is included in the new assembly, rearranged, and slightly extended. All together the assembly graph in figure 1 is likely to represent complete *Z. marina* mitogenome with the summary length of the edges of 187,048 bp resulting in a considerably compact but within the range of plant mitochondrion genomes.

Postassembly polishing and validation of the mitogenome sequence were performed using Illumina reference short reads, which were obtained from Olsen et al. (2016) (SRR3926352). The aligned short reads were used to correct short indels, deletions, and mismatches in each selected PacBio contig. Low-quality contig ends were omitted from the final genome reconstruction. Short-read coverage of the assembled mitogenome was calculated directly by SAMtools from the raw read mapping performed by Burrows-Wheeler aligner – maximal exact matches (BWA-MEM) with default parameters (Li 2013; Danecek et al. 2021). To overcome edge effects on coverage, Illumina reads were aligned to two different linear forms of the joined mitochondria assembly with nonrepeated break points of the circles. Then the final coverage was calculated per position as maximum coverage of the corresponding position in two shuffles. The continuous coverage of the assembled mitogenome by the Illumina short reads served as an additional confirmation of the assembly accuracy.

### PacBio Reads for Isoform Validation

Recombination activity of repeats was quantified with the use of PacBio long reads. Potential recombination sites were identified by aligning the assembled mitochondrion to itself by BLAST with the minimum repeat length of 50 bp, minimum identity 80%, and minimum distance between the two copies of 2000 bp. A total of 380 direct and 245 inverted repeat pairs were identified. A recombination database was constructed, containing four possible combination of 1000 bp flanks for each repeat pair: M1, M2—flank composition as they are in the assembly master circles; R1, R2—recombined versions. All PacBio reads were aligned to the recombination database by BLAST with the minimum identity 80% and minimum flank coverage 80%. The proportion of reads aligned to R1 or R2 was used as an estimate of the recombination activity of a particular repeat pair. Because of a certain region complexity, we additionally marked repeats, which are nested in Rep1 or Rep2 and therefore might show a misleading recombination signal. For the majority of repeat pairs, the proportion of recombined configurations was below 2% but above zero, hence, corresponding PacBio reads are likely to originate from substoichiometric forms.

To assess the genome segments (**a**…**i**) combination frequencies, as shown in figure 2*B*, we used long PacBio reads that were covered by a single alignment with at least 60% of each of the two 500 bp flanks of a genome segment junction. Another approach engaging PacBio long reads for isoform validation was an alignment of reads to all reconstructed isoforms with further filtration for unambiguously defined read-isoform pairs. Such pairs occur when a PacBio read covers a unique sequence that univocally defines a specific isoform. The minimum length of a unique sequence to confirm an isoform varies significantly, therefore the numbers of sustaining reads are comparable only among iso1*–isoI–isoIV group (24,730 bp minimum length required) and between iso2 and isoII (7,121 bp minimum length required). Corresponding numbers of PacBio reads confirming comparable isoforms reflect their relative abundance in the reference sample. For isoforms iso1, isoIII, and isoV, the minimum length of a PacBio read to cover an unambiguous isoform region to be properly paired is over 39,579 kbp, which dramatically decreases the probability to find at least one suitable PacBio read, thereby explaining the absence of such reads in our dataset.

### Mitogenome Annotation

Mitogenome annotation was performed by tBLASTn of protein sequences of other known mitogenomes within order Alismatales including *Z. marina* previous assembly (Petersen et al. 2017), tRNAs were additionally annotated by Mitofy (tRNAscan-SE) (Lowe and Eddy 1997; Alverson et al. 2010) and ARAGORN (Laslett 2004). In comparison with the previous assembly, the annotation of *cox2* gene appeared as a complete open reading frame and eight additional tRNA genes were identified. Mitochondrial DNA regions shared with nuclear chromosomes (NUMTs) and chloroplasts (mtptDNA) were detected by BLAST with minimum identity 85% and maximum *e*-value 1 × 10^−10^.

### Worldwide Population Dataset and Finnish Clone Dataset Analysis

Data of Illumina sequencing of 163 samples from 16 populations were retrieved from a previous study of *Z. marina* populations (Yu et al. 2023). Raw reads were mapped by BWA-MEM with default parameters to 67 core nuclear single copy genes longer than 3000 bp to calculate target coverage of the sequencing per single replicon which ranges from × 2.5 to × 55.5. Similarly, raw reads were aligned to the assembly mitogenome graph genome content. Genome segments were defined as unbreakable parts of the mitochondrion—nine in total counting Rep1 and Rep2 only once (fig. 2). Genome segment copy numbers were assessed as median coverage of non-NUMT and non-mtptDNA positions normalized on one replicon target coverage of the corresponding sample. The target sequencing coverage was assessed by the mean coverage of 67 nuclear genes that are found in the genome in a single copy and are over 3 kbp long, divided by two (because *Z. marina* has a diploid nuclear genome). For the isoform stoichiometry calculation and the sample pair analysis, only non-WAS samples with the minimum coverage of segment **b** (Rep2) of 40 × were selected, including 132 samples in total. Samples of the Finnish clone meristematic region (*n* = 24) and developed leaf samples (*n* = 5) were analyzed in the same way as the worldwide population dataset.

## Supplementary Material

evad167_Supplementary_Data

## Data Availability

Genome assembly is available on the ORCAE platform (https://bioinformatics.psb.ugent.be/gdb/zostera/). The PacBio long-read sequencing data are available at the NCBI SRA database (BioProject: PRJNA701932; SRA accessions: SRX10442518–SRX10442525). The Illumina whole-genome sequencing of the inner leaf base is available at the NCBI SRA database (BioProject: PRJNA41721; SRA accession: SRR3926352) for the reference sample sequencing, at the NCBI SRA database (BioProject: PRJNA462974; SRA accessions: SRP193415–SRP227670) for the worldwide population dataset, and at the NCBI SRA database (BioProject: PRJNA557092; SRA accessions: SRX6631885–SRX6631894, SRX6631898–SRX6631911) for the Finnish clone dataset. The Illumina whole-genome sequencing of leaf tissues is available at the NCBI SRA database (BioProject: PRJNA972892; SRA accessions: SRR24578335–SRR24578339). The newly assembled mitochondrial genome is available in the GenBank database (accessions: OR336317–OR336318).
