# Supplementary material for: Worldwide Population Genomics Reveal Long-Term Stability of the Mitochondrial Genome Architecture in a Keystone Marine Plant
Source: Genome Biol Evol. 2023 Sep 14;15(9):evad167. doi: 10.1093/gbe/evad167 (PMC10538256; doi:10.1093/gbe/evad167)
Supplement: evad167_Supplementary_Data [file evad167_supplementary_data.pdf]

Worldwide population genomics reveal long-term stability of the mitochondrial genome architecture in a keystone marine plant

Supplementary information

Table of Contents

Supplementary information 1

Supplementary Tables 2

Supplementary Table S1: *Z. marina* mitochondrial genome segments...............................2

Supplementary Table S2: Long read alignments for isoform confirmation..........................3

Supplementary Figures 4

Supplementary Fig. S1: Recombination activity analysis of all large and intermediate size repeat pairs within the *Z. marina* mitogenome. 4

Supplementary Fig. S2: Absolute copy number of genome segments **a…i** in individual samples. 5

Supplementary Fig. S3: Intrachromosomal duplication detected in all WAS samples. 6

Supplementary Fig. S4: The transformation of the nine genome segment stoichiometry into the isoform stoichiometry...7

References...8

Supplementary Tables

Supplementary Table S1: *Z. marina* mitochondrial genome segments. Genome segment names and colors correspond to Figure 2; segment length (bp) is the corresponding total length of the sequence; non-shared segment length (bp) is the number of non-NUMT and non-mtpt positions which were incorporated in the copy number calculation; encoded genes are presented in accordance with the new annotation available in the GenBank database (accessions: OR336317-OR336318). *Z. marina* is characterized by a small mitochondrial genome as predicted in Petersen et al. 2017 similar to the compact nuclear genome (Olsen et al. 2016; Ma et al. 2021). However, a uniform way of calculating the mitogenome size in case of a multipartite architecture is not settled. A simple length summary of the primary chromosomes isoI-V yields a total size of 449,541bp, however using this formulation, specific genome segments are counted multiple times. If calculated as total length of the master circles only, excluding the subgenomes or alternative rearrangements (Alverson et al. 2011), the total mitochondrial genome size is 336,352bp, which is the length of iso1 and iso2 together (that similarly includes repeated genome content). A better representation of the mitogenome size should be the total length of the optimal assembly graph (Varré et al. 2019), namely 187,048bp, or in the extreme case, 178,508bp, if each genome segment **a**…**i** is counted once.

Genome segment	Segment color on Figure 2	Segment length (bp)	Non-shared segment length (bp)	Genes	
				Protein coding	RNA
**a**	red	3,424	3,309	*cob*, nad4L, atp6**	-
**b**	yellow	3,695	2,844	*atp6**	*trnE, trnQ*
**c**	green	46,792	37,493	*nad6, nad7, ccmFc, cox1, nad9, ccmFn, rps7*	*trnD, trnMf, rrn5, rrn18*
**d**	pink	4,845	3,102	-	*trnC*
**e**	sand	16,188	8,160	*nad4, atp4*	*trnT*
**f**	blue	70,665	31,845	*nad5*, ccmC, nad1*, matR, atp9, cox3, nad3, atp1, cox2**	*trnR, trnN, trnL-cp, trnW, trnT, rrn26, trnM*
**g**	violet	2,947	1,430	*cox2*, mttB*	*trnI-cp, trnA*
**h**	marine	27,613	25,030	*ccmB, nad5*, nad2, atp8, nad1**	*trnC, trnI(trnM)*
**i**	grey	2,339	2,174	*nad1*, cob**	-

* Genes partially overlapping with the genome segment.

Supplementary Table S2: Long read alignments for isoform confirmation. The unique genome segment combination is the minimum in length order of genome segments represented exclusively in the corresponding isoform; the minimum read length (bp) is the minimum long read length to confirm the corresponding isoform; the number of aligned PacBio reads is the number of reads which confirmed the corresponding chromosome being aligned to the identified unique genome segment combination.

Isoform	Unique genome segment combination	Minimum read length (bp)	Number of aligned PacBio reads
iso1	**g-d-h-a-b-c**	39,579	0
iso2	**i-a-b-c**	7,121	97
iso1*	**g-d-e-b-c**	24,730	10
isoI	**c-d-e-b-c**	24,730	5
isoII	**i-a-b-f**	7,121	93
isoIII	**c-d-h-a-b-c**	39,579	0
isoIV	**g-d-e-b-f**	24,730	3
isoV	**f-g-d-h-a-b-f**	42,526	0

Supplementary Figures

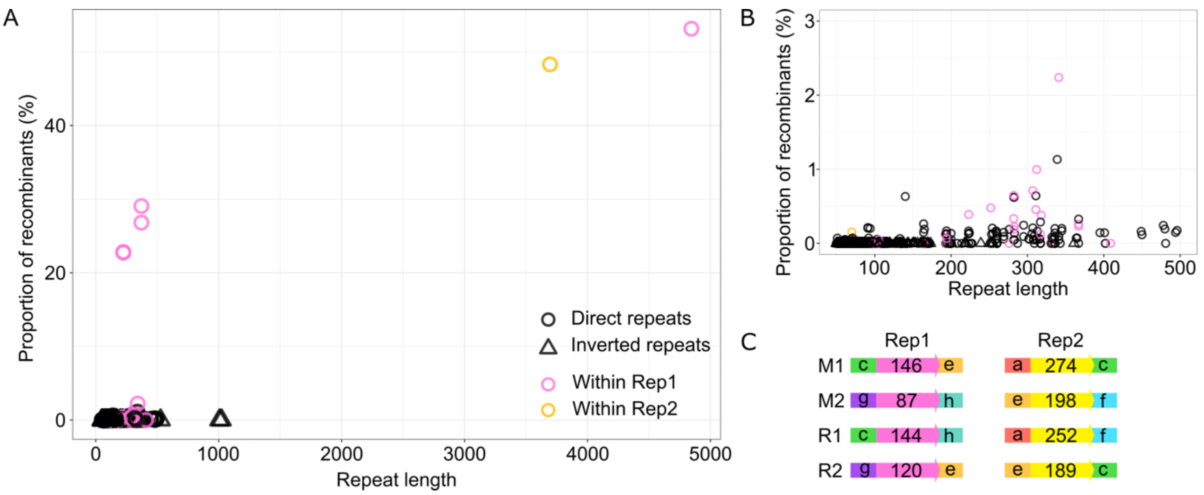

Supplementary Fig. S1: Recombination activity analysis of all large and intermediate size repeat pairs within the *Z. marina* mitogenome. a, Recombination activity of all intermediate size and large repeat pairs. Circles indicate direct repeats, triangles – inverted repeats. Repeats nested in (or equal to) Rep1 and Rep2 are colored in pink and yellow correspondingly, such intermediate size repeats might show a signal which is a reflection of the Rep1 and Rep2 recombinational activity and therefore cannot be taken into account. b, A zoom into the bottom-left part of the graph on a, with multiple repeats demonstrating weak recombinational activity and likely referring to the substoichiometric reservoir. c, The master (M1, M2) and alternative (R1, R2) flank compositions of the repeats Rep2 and Rep1 with the numbers of PacBio reads supporting the corresponding flank composition.

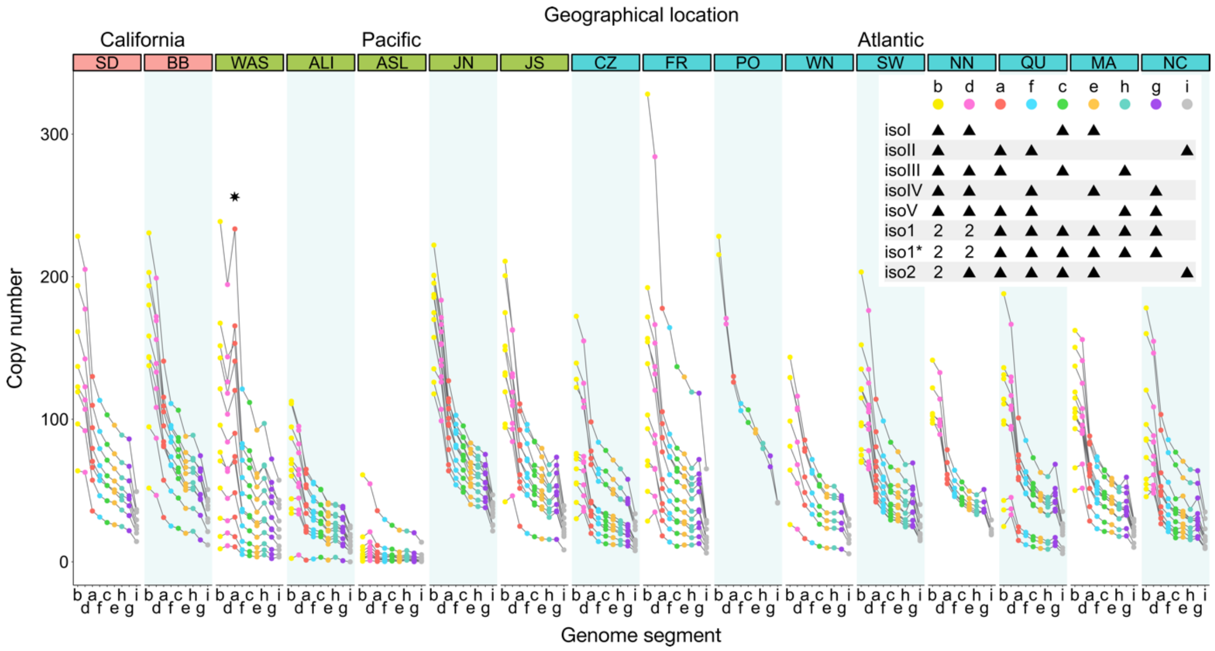

Supplementary Fig. S2: Absolute copy number of genome segments a…i in individual samples. The samples are grouped by location of sampling, locations are subdivided into three phylogenetically distant groups: Californian, Pacific, and Atlantic (Yu et al. 2023). Segment copy number is depicted by dots (colored according to Figure 2); the dots are connected by grey lines within individual samples. An increased genome segment **a** copy number due to duplication in the WAS samples is highlighted by a star. Segment composition of the main genome isoforms is shown in the legend (top right) where black triangles show the presence of segments in the corresponding isoform.

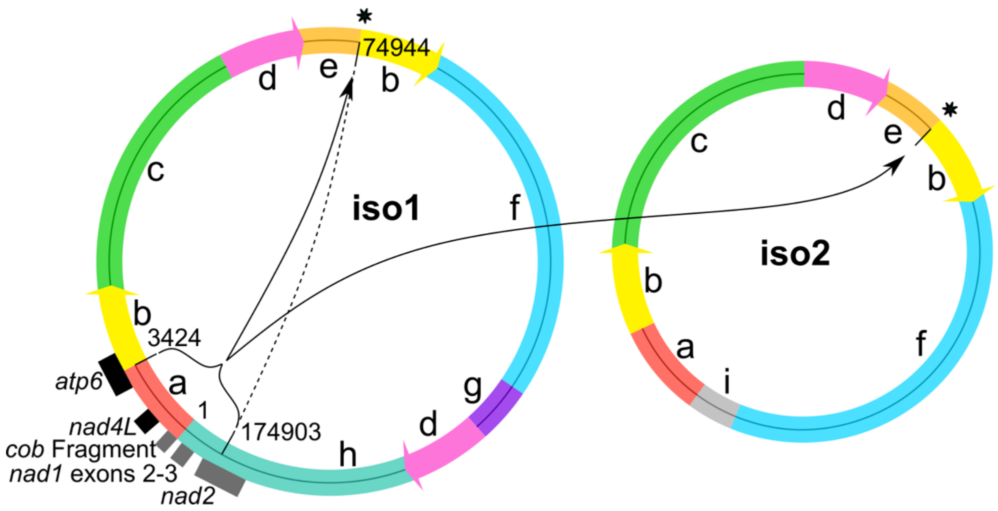

Supplementary Fig. S3: Intrachromosomal duplication detected in all WAS samples. Short Illumina reads aligned to the reference genome show no linkage between segments **e** and **b** in all WAS samples, therefore pointing towards a breakpoint between **e** and **b** in all isoforms (marked with a star). Instead, an additional contact was identified between the position 174903 in segment **h** and the beginning of segment **b** (dashed line). Given the doubled coverage of the region 174903-3424 we concluded that this region was duplicated in between segments **e** and **b** in both master circles iso1 and iso2 and all their derivatives that have consequent segments **e** and **b** in other samples (black arrows). Grey rectangles show all genes that partially overlay with the duplicated region. The black rectangles indicate *nad4L* and *atp6* genes duplicated in a complete form proposing a possible increase of their expression level.

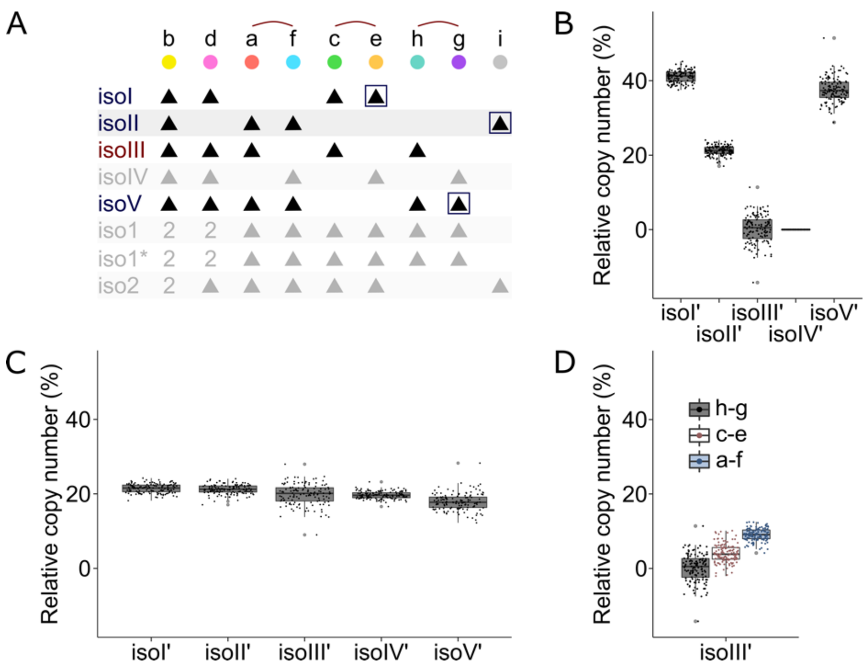

Supplementary Fig. S4: The transformation of the nine genome segment stoichiometry into the isoform stoichiometry. a, The scheme of the nine genome segment distribution among the main genome isoforms when fixing the reversible recombination reactions to zero iso1, iso1*, iso2, and isoIV products. The squares indicate genome segments that univocally represent subgenome isoI', isoII', and isoV' copy number. The brown arcs indicate simple subtractions for the isoIII' copy number calculation. b, Relative copy number of the subgenomes isoI'-V' after fixing all reversible recombination reactions calculated for 132 reliable samples. The isoIII' copy number is calculated as the **h-g** subtraction. c, Relative copy number of the subgenomes isoI'-V' estimated as in b, with an additional assumption of copy number equality of subgenome pairs isoI-isoV and isoIII-isoIV. d, Relative copy number of the subgenome isoIII' after fixing all reversible recombination reactions calculated as **h-g**, **c-e**, and **a-f** subtractions. The three methods provide significantly different results with Kruskal-Wallis test p-value below 2.2e-16s.

References

Alverson AJ, Rice DW, Dickinson S, Barry K, Palmer JD. 2011. Origins and Recombination of the Bacterial-Sized Multichromosomal Mitochondrial Genome of Cucumber. *Plant Cell* 23:2499–2513.

Ma X, Olsen JL, Reusch TBH, Procaccini G, Kudrna D, Williams M, Grimwood J, Rajasekar S, Jenkins J, Schmutz J, et al. 2021. Improved chromosome-level genome assembly and annotation of the seagrass, Zostera marina (eelgrass). *F1000Res* 10:289.

Olsen JL, Rouzé P, Verhelst B, Lin Y-C, Bayer T, Collen J, Dattolo E, De Paoli E, Dittami S, Maumus F, et al. 2016. The genome of the seagrass Zostera marina reveals angiosperm adaptation to the sea. *Nature* 530:331–335.

Petersen G, Cuenca A, Zervas A, Ross GT, Graham SW, Barrett CF, Davis JI, Seberg O. 2017. Mitochondrial genome evolution in Alismatales: Size reduction and extensive loss of ribosomal protein genes.Mishmar D, editor. *PLoS ONE* 12:e0177606.

Varré, D'Agostino, Touzet, Gallina, Tamburino, Cantarella, Ubrig, Cardi, Drouard, Gualberto, et al. 2019. Complete Sequence, Multichromosomal Architecture and Transcriptome Analysis of the Solanum tuberosum Mitochondrial Genome. *IJMS* 20:4788.

Yu L, Khachaturyan M, Matschiner M, Healey A, Bauer D, Cameron B, Cusson M, Emmett Duffy J, Joel Fodrie F, Gill D, et al. 2023. Ocean current patterns drive the worldwide colonization of eelgrass (Zostera marina). *Nat. Plants* [Internet]. Available from: https://www.nature.com/articles/s41477-023-01464-3
